# Evaluating Prevalence of Preterm Postnatal Growth Faltering Using Fenton 2013 and INTERGROWTH-21st Growth Charts with Logistic and Machine Learning Models

**DOI:** 10.3390/nu17101726

**Published:** 2025-05-20

**Authors:** Ioanna Kakatsaki, Nicolina Hilda Anagnostatou, Theano Roumeliotaki, Eleftherios Panteris, Theodoros Liapikos, Styliani Papanikolaou, Eleftheria Hatzidaki

**Affiliations:** 1 Department of Neonatology and NICU, University General Hospital of Heraklion, School of Medicine, University of Crete, 70013 Crete, Greece; joanne_k1991@hotmail.com (I.K.); nicolehilda@gmail.com (N.H.A.); eleftherios.panteris@gmail.com (E.P.); stylpapa12@gmail.com (S.P.); 2 Clinic of Preventive Medicine and Nutrition, Division of Social Medicine, School of Medicine, University of Crete, 70013 Crete, Greece; roumeliot@uoc.gr; 3 Department of Chemistry, Aristotle University of Thessaloniki, 54124 Thessaloniki, Greece; theoliapikos@gmail.com

**Keywords:** prematurity, restricted growth, SGA, PGF, extremely preterm, very preterm, INTERGROWTH-21st, Fenton 2013, machine learning

## Abstract

**Background/Objectives:** Postnatal growth faltering (PGF) significantly affects premature neonates, leading to compromised neurodevelopment and an increased risk of long-term health complications. **Methods:** This retrospective study at a level III NICU of a tertiary hospital analyzed 650 preterm neonates born before 33 weeks. Postnatal growth was evaluated using the Fenton2013 and INTERGROWTH-21st growth charts, with changes in weight z-scores from birth to discharge classified as normal growth (ΔZ ≥ −1), non-severe PGF (−2 ≤ ΔZ < −1), and severe PGF (ΔZ < −2). **Results:** Mean gestational and postmenstrual age at discharge was 30 weeks (SD 1.9) and 37.1 weeks (SD 2.7), respectively. Fenton2013 growth curves revealed a higher prevalence of non-severe and severe PGF (43% and 14.6%) compared to INTERGROWTH-21st (24.5% and 10.3%). A more rapid establishment of full enteral feeds was strongly associated with reduced severe PGF prevalence in both growth charts (*p* < 0.001), as was shorter hospitalization. Late-onset sepsis was associated with an increased risk of severe PGF, while being small for gestational age (SGA) was protective against severe PGF across both growth charts (*p* < 0.001). A trend of decreasing PGF prevalence was noted over the study years, most probably attributed to the implementation of updated nutritional guidelines. Interestingly, when machine learning classification models were evaluated in our Greek cohort, a notable decline in predictive accuracy depending on the growth standard applied was observed. **Conclusions:** Our study highlights the need for standardizing PGF definition in an attempt to enhance nutritional management and further investigate the long-term impacts of nutritional interventions on growth, neurodevelopment, and overall health outcomes.

## 1. Introduction

Advances in perinatal care have undeniably improved the survival rates of neonates, particularly those born at extremely low gestational ages [1,2]. Nonetheless, extrauterine growth restriction (EUGR) or postnatal growth faltering (PGF) using the alternative term remains a major problem seriously affecting premature neonates [3,4]. Besides poor somatic growth, those neonates face compromised neurodevelopment and an increased risk of chronic health issues such as hypertension, diabetes, and cardiovascular diseases [5,6,7,8,9,10]. Considering that suboptimal postnatal growth is potentially preventable, it emphasizes the critical importance of accurate growth assessment in order to ensure optimal development and well-being in preterm infants [11].

Premature neonates experience suboptimal growth during their early postnatal period, often referred to as their ‘third trimester’ of life. This is influenced by multiple factors besides gestational age, including being small for gestational age (SGA), high energy demands due to rapid brain growth and complications of prematurity, and suboptimal feeding practices [12,13,14,15,16]. As a result, the initial period after birth is characterized by a mismatch between energy requirements and actual nutritional intake of these preterm neonates, being much more pronounced for the sickest neonates among them [17,18].

The prevalence of PGF among preterm infants has been observed to be alarmingly high, necessitating urgent attention to address this issue [19]. However, existing studies are difficult to compare due to the absence of a consistent definition, the formerly used definition of extrauterine growth restriction (EUGR), and the considerable variation in growth assessment charts being employed. The cross-sectional definition of EUGR relies on z-scores or percentiles evaluated at specific timepoints, such as 36–40 weeks postmenstrual age (PMA) or at discharge, while longitudinal EUGR is defined as a decrease of 1 or 2 z-scores from birth to the aforementioned time points [20]. According to the latest position paper by ESPGHAN [21], the term “postnatal growth faltering” is emerging to describe insufficient postnatal growth and its usage is increasingly prevalent in recent studies [22,23]. The term “postnatal growth faltering” better reflects variations in z-scores and in the literature is defined by longitudinal criteria as a decrease of 1 or 2 standard deviations (SDs) from birth to specific time points, such as 36–40 weeks PMA or at discharge [22]. This longitudinal approach is favored for evaluating inadequate growth and detecting infant malnutrition, as it reflects growth trends over time and facilitates the earlier identification of PGF [5,20].

Growth charts are percentile curves showing the distribution of selected body measurements, such as weight, length, and head circumference, from the neonatal period through adolescence. Understanding the methodology and the population used for their construction is crucial for effective utilization [24]. The Fenton 2013 growth charts, derived from a meta-analysis of six extensive population-based surveys encompassing almost four million births in developed countries, are widely used. These charts were developed by observing fetal growth patterns in the womb, enabling the assessment of nutritional status during intrauterine development [25]. However, they have limitations in accounting for postnatal weight changes in preterm infants, highlighting the substantial differences between prenatal and postnatal growth, influenced by diverse environmental and nutritional factors [26]. The INTERGROWTH-21st Project is a prospective, longitudinal, multi-ethnic study characterized by stringent selection criteria, focusing on describing the optimal rather than average growth of preterm infants and incorporates preterm newborns who experienced an uncomplicated intrauterine life and infancy. The INTERGROWTH-21st Project introduced the INTERGROWTH-21st Postnatal Growth Standards to monitor the postnatal growth of preterm infants, complementing the existing WHO Child Growth Standards up to 64 weeks [27]. In 2016, the inclusion of preterm neonates from pathological pregnancies broadened the dataset, leading to the creation of the INTERGROWTH-21st Very Preterm Growth Charts [28].

Currently, there is no consensus regarding which growth assessment tool is suitable for monitoring postnatal growth of preterm infants or defining EUGR or PGF [20,21,29]. In the literature, longitudinal EUGR or PGF has been shown to better predict somatic growth outcomes at 24–30 months compared to cross-sectional EUGR [30]. However, a definitive conclusion regarding its predictive value for neurodevelopmental outcomes has not been established [31,32,33,34]. In our study, we chose to use the dynamic longitudinal definition for assessing PGF, recognizing the benefits of continuous evaluation. The primary aim of the present study was to describe the difference of PGF prevalence among preterm infants born before 33 weeks of gestational age in a level III Neonatal Intensive Care Unit (NICU) according to the two growth charts most used in clinical practice (Fenton 2013 and INTERGROWTH-21st). The secondary aim was to identify neonatal and nutritional factors associated with PGF.

## 2. Methods

A clinical retrospective study was designed and carried out at the Department of Neonatology/Neonatal Intensive Care Unit of the University General Hospital of Heraklion (PAGNI), a level III NICU in a tertiary hospital with an annual admission rate of nearly 600 infants. A total of *n* = 738 preterm neonates born before 33 weeks were eligible to participate in the study during a period of 15 years, from January 2008 to December 2022. Exclusion criteria were major congenital malformations and genetic syndromes. Further sample reductions were made due to death before hospital discharge (*n* = 29), transfer to another hospital before discharge (*n* = 1), unattainable data in the medical records (*n* = 39), and being born in another hospital (*n* = 16). From the initial population, *n* = 650 preterm newborns were finally studied.

Clinical, nutritional, and growth data were retrospectively collected from hospital records. The study received approval from the University General Hospital of Heraklion Bioethics Committee Protocol Number 17946/07-09-2022 and the University General Hospital of Heraklion PAGNI Board of Directors decision Number 1143/19-10-2022, which waived consent for retrospective data access to medical records, and all data were anonymized before analysis.

The primary outcome was to assess the growth pattern of weight at two time points—birth and NICU discharge—and to identify which neonates exhibited PGF based on the two different growth charts. Birth weight, gestational age, and the biological sex of the neonate were recorded within 1 h after delivery, as was the weight at discharge; weight was measured using adapted patient incubator and infant digital weighing scale. Gestational age (GA) was determined according to either the last menstrual period or an early prenatal ultrasound or calculated directly in case of in vitro fertilization. Postmenstrual age at discharge was calculated as gestational age plus chronological age in weeks [35]. Growth data were plotted on both the Fenton 2013 growth charts [25] and the INTERGROWTH-21st Very Preterm Growth Charts and INTERGROWTH-21st Postnatal Growth Standards for weight at birth [28] and at discharge, respectively [36]. PGF was assessed based on the longitudinal change in weight z-score (ΔZ) from birth to hospital discharge. Infants were categorized into three groups according to the magnitude of z-score decline. Normal growth was defined as a change in weight z-score greater than or equal to −1 standard deviation (ΔZ ≥ −1), non-severe PGF was marked by a decrease in weight z-score between −1 and −2 standard deviations (−2 ≤ ΔZ < −1), and severe PGF was marked by a loss exceeding 2 standard deviations of weight z-score from birth to discharge (ΔZ < −2) [4,21,22,23]. Evaluated nutritional factors in our study were the day of life that enteral (EN) and parenteral nutrition (PN) were initiated, the time to reach full feeds, and the duration of parenteral nutrition. It should be noted that the study took place during a period of 15 years, so the feeding practices in NICUs have been changing, adapting to current international recommendations [21,37,38,39,40,41]. Traditionally, a conservative feeding approach post-birth involved delayed feeding initiation, with feeds introduced after four days, and a gradual increase in feeding volume at a maximum rate of 24 mL/kg/day. However, evolving practices have shifted towards a more liberal strategy. This approach includes the initiation of PN on the first day of life and the early introduction of enteral feeding within 24–48 h after birth, with gradual advancement based on the infant’s tolerance, aiming to achieve full enteral feeds in a shorter period. Enteral feeding, whether with expressed own mother’s milk or specialized formula for preterm infants, was initiated concurrently with a reduction in parenteral nutrition. Breastfeeding was fortified upon reaching an enteral feeding volume of 100 mL/kg/day.

Additional information recorded for each infant included multiple gestation, type of delivery, length of hospitalization, duration of ventilation (invasive and non-invasive), oxygen administration, and morbidities related to prematurity, such as bronchopulmonary dysplasia (BPD) [42], apnea of prematurity and its associated treatment [43,44], early-(<72 h) and late-onset (>72 h) culture proven or suspected sepsis [45], hemodynamically significant patent ductus arteriosus (hsPDA) requiring treatment [46], necrotizing enterocolitis (NEC) staged according to the modified Bell criteria (stage IIIB) [47,48], anemia requiring at least one blood transfusion [49,50], retinopathy of prematurity (ROP) (Stage II or greater) [51,52], cystic periventricular leukomalacia (PVL), and intraventricular hemorrhage (IVH) grade I–IV based on ultrasound diagnosis [53,54].

### Statistical Analysis

Firstly, the distribution of the independent and outcome variables of interest was examined by estimating descriptive statistics. Continuous variables were summarized as mean ± standard deviation (SD) if normally distributed or as median with interquartile range (IQR) if not; the IQR, defined as the range between the 25th and 75th percentiles, reflects the central 50% of the data. Cochran’s test was used to test for equality of proportions between the different growth charts. Normality of distribution was tested with the Shapiro–Wilk statistic indicating the appropriateness of non-parametric testing. Thus, bivariate associations between continuous variables were examined using Spearman’s correlation coefficient and Mann–Whitney or Kruskal–Wallis tests for categorical variables. Finally, Pearson’s chi-square test was used to test the prevalence of categorical variables.

Initially, logistic regression models were employed in order to identify significant clinical and nutritional factors associated with the risk of severe PGF. Multivariate models included the factors found to be correlated (*p* < 0.05) at the bivariate comparisons previously described and to have expected frequencies above 5% in all three levels of the outcome variables (i.e., PGF by Fenton and INTERGROWTH-21st). Thus, clinical factors NEC, PDA, ROP, and PVL were not further examined in multivariate analysis due to the abovementioned data restrictions. Multicollinearity between independent variables was also assessed using the variance inflation factor (VIF), resulting in the exclusion of mechanic and non-invasive ventilation from regression models. Effect modification by sex was explored by stratifying the sample. Additional sensitivity analysis was performed using multinomial logistic regression models in order to examine the effects of the various clinical and nutritional factors with the severity of PGF. All statistical testing was based on two-tailed hypotheses with 5% significance level. The statistical software used was Stata v13 (Stata Corp, College Station, TX, USA).

Additional machine learning techniques were applied to the dataset, utilizing a combination of supervised and unsupervised approaches to analyze preterm populations. Infants were first grouped by gestational age into three categories: extremely preterm (<28 weeks), very preterm (28–31^6/7^ weeks), and moderately preterm (32–32^6/7^ weeks). Separately, they were also categorized based on their birth weight in relation to gestational age into three growth categories: SGA: small for gestational age, AGA: appropriate for gestational age, LGA: large for gestational age. These classifications served as a three-class target variable for sample classification.

All machine learning algorithms used in this study, including Extreme Gradient Boosting (XGBoost), HDBSCAN (Hierarchical Density-Based Spatial Clustering of Applications with Noise), Hierarchical Clustering, and K-Means, were implemented using Python v. 3.81. Specifically, the scikit-learn library was utilized for K-Means, Hierarchical Clustering, and other preprocessing steps, while XGBoost and HDBSCAN were implemented using the XGBoost and HDBSCAN libraries, respectively.

For classification, XGBoost [55], an optimized gradient boosting framework recognized for its efficiency and scalability, was employed. We implemented a double-nested cross-validation (CV) approach to develop and evaluate an XGBoost classifier while mitigating overfitting risks [56]. The outer CV consisted of 10 folds for model performance assessment, while the inner CV utilized 7 folds for hyperparameter optimization. For each outer CV iteration, samples were split into training and test sets. Within each training set, a randomized grid search was performed across the inner seven-fold CV to identify optimal hyperparameters. The hyperparameter space included the number of estimators, maximum tree depth, learning rate, minimum loss reduction for partition, L1 regularization, and L2 regularization. The best-performing hyperparameter configuration from each inner CV was used to train a model on the corresponding outer training fold and evaluate performance on the outer test fold. To address any class imbalance, sample weighting was applied, adjusting the contribution of each class during training based on its relative frequency in the dataset. Feature importance values were extracted after each outer CV iteration. The final variable importance was calculated as mean ± standard deviation across all outer CV iterations, providing robust estimates of each predictor’s contribution to the model while accounting for variability in the feature selection process. This methodology ensured unbiased performance evaluation while simultaneously optimizing the model and quantifying the stability of feature importance.

Prior to model training, the dataset underwent preprocessing to ensure compatibility with machine learning algorithms. Numerical features were normalized using z-score standardization, transforming each variable to have zero mean and unit variance. The categorical dependent variables were transformed using one-hot encoding, with the first category dropped to avoid multicollinearity. These preprocessing steps were applied consistently within the machine learning pipeline to prevent data leakage during cross-validation.

Model performance was evaluated using multiple metrics to ensure a comprehensive assessment of classification effectiveness. These included the Weighted F1-score, which accounts for class imbalance by weighting the F1-score of each class based on its frequency; Cohen’s Kappa Score, which measures agreement between predictions and true labels while correcting for chance agreement; Matthews Correlation Coefficient (MCC), a robust metric for imbalanced classification that considers true positives, true negatives, false positives, and false negatives; and Macro-Averaged Area Under the Receiver Operating Characteristic Curve (AUC Macro Avg), which evaluates the model’s ability to distinguish between classes across all classification thresholds. These metrics provided a balanced and reliable evaluation, particularly in the presence of class imbalance, ensuring that the model’s predictive performance was not biased toward majority classes (Supplementary Table S1).

For unsupervised analysis, clustering techniques were applied, including Hierarchical Clustering [57,58], K-Means [59], and HDBSCAN [60,61]. These methods were employed to uncover underlying patterns and group the data based on inherent similarities, without reference to the target variable. The clustering results were evaluated using several metrics such as Normalized Mutual Information (NMI), which measures the amount of information shared between the clustering results and the true labels, adjusted for randomness; the Adjusted Rand Index (ARI), which assesses the similarity between the predicted clusters and the ground truth labels, adjusting for chance grouping; the Silhouette Score, which evaluates the quality of the clusters by measuring how similar each point is to its own cluster compared to other clusters, with higher values indicating better-defined clusters; and the Davies–Bouldin Index, which measures the average similarity ratio of each cluster with the one that is most similar to it, with lower values indicating better separation between clusters. Clustering results were visualized using t-distributed Stochastic Neighbor Embedding (t-SNE), a dimensionality reduction technique that allowed for the visualization of high-dimensional data in two or three dimensions. Additionally, dendrograms were generated for hierarchical clustering to illustrate the hierarchical relationships between clusters. These visualizations provided valuable insights into the dataset’s structure and complemented the supervised classification task. The combination of supervised and unsupervised learning approaches allowed for both accurate classification and in-depth data exploration, contributing to a comprehensive understanding of the dataset’s patterns and relationships.

The evaluation of the machine learning models began by establishing baseline performance using classification into clearly defined gestational-age groups. This initial step confirmed that the algorithms effectively utilized clinical and nutritional factors to accurately classify neonates within these gestational categories. After demonstrating robust performance with these standard classifications, the same models were then applied to classify infants based on the Fenton 2013 and INTERGROWTH-21st growth curves, which represent alternative and potentially more complex methods of stratification.

## 3. Results

The study population characteristics are presented in Table 1. The sample was well balanced between male and female infants, with a slightly higher percentage of males (53.8%). Mean gestational age was 30 weeks (SD 1.9), whereas 12.3% of infants were born before 28 weeks, 58.3% between 28 and 32 weeks, and 29% between 32 and 33 weeks of gestation. Postmenstrual age at discharge had a mean value of 37.1 weeks (SD 2.7).

**Figure 1:**
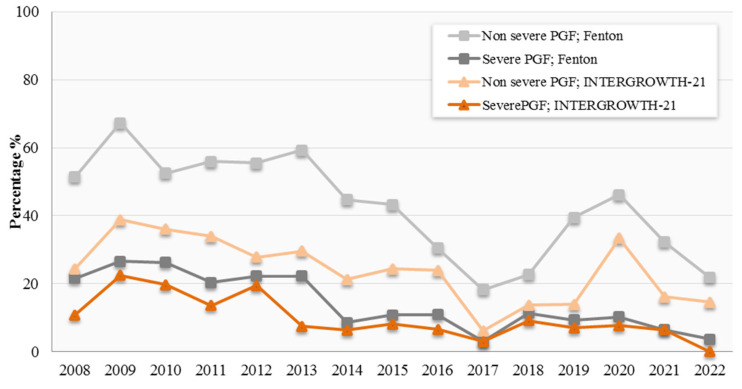
Prevalence of postnatal growth faltering (PGF) by year of study.

The prevalence of PGF varied across the years of study, with a decreasing trend observed by the graphical representation (Figure 1). The prevalence of non-severe PGF observed from the Fenton 2013 growth curves was recorded at 43%, compared to 24.5% through the INTERGROWTH-21st growth curves. Additionally, the incidence of severe PGF was reported at 14.6% in the Fenton 2013 growth curves, compared to 10.3% in the INTERGROWTH-21st. Proportions of PGF were significantly different (*p* < 0.001) between the Fenton and INTERGROWTH-21st growth charts, where the prevalence of both non-severe and severe PGF using Fenton 2013 was higher.

Bivariate tests of unadjusted comparisons of measured clinical and nutritional factors with the prevalence of PGF revealed strong associations (Table 2). Infants born extremely preterm (<28 weeks of gestation) were more likely to experience severe PGF (48.7% and 38.5%, by Fenton 2013 and INTERGROWTH-21st, respectively) compared to those born between 28 and 32^6/7^ weeks of gestation. On the contrary, SGA infants had significantly lower percentage of severe and non-severe PGF (Fenton 2013: *p* = 0.018 and INTERGROWTH-21st: *p* = 0.018). Median duration of hospitalization was more than double for severe PGF [Fenton 2013: 76.5 (34.0); INTERGROWTH-21st: 83.5 (35.0) days] compared to infants without PGF [Fenton 2013: 36.0 (18.0); INTERGROWTH-21st: 36.5 (19.0) days]. Similar findings were observed for almost all clinical factors recorded, as shown in corresponding Table 2.

Finally, nutritional factors were also associated with the severity of PGF, where median (IQR) duration of parenteral nutrition was 6 (10), 7 (12), and 19 (26) days by Fenton 2013 and 6 (11), 9 (11), and 22.5 (25) days by INTERGROWTH-21st for infants without PGF, with non-severe PGF, and with severe PGF, respectively (*p* < 0.001). Enteral nutrition was initiated later for infants exhibiting non-severe PGF (fourth day of life) and infants with severe PGF (sixth or seventh day of life) compared to infants without PGF (second day of life) irrespective of the chart used. Furthermore, full enteral nutrition was achieved on the 9th day of life for infants without PGF, 12th (Fenton 2013) or 16th (INTERGROWTH-21st) day of life for infants with non-severe PGF, and 25th (Fenton 2013) or 27th (INTERGROWTH-21st) day of life for infants with severe PGF (*p* < 0.001). Similar findings were obtained from the comparison of clinical and nutritional factors with severe PGF presented in Supplementary Table S2.

The assessed effect of the recorded clinical and nutritional factors on the risk of severe PGF, using both reference charts, is presented in Table 3. The factor with significant protective effect was SGA (Fenton 2013: OR = 0.11, 95% CI 0.01–0.92; INTERGROWTH-21st: OR = 0.10, 95% CI 0.02–0.60). The prevalence of severe PGF was higher for each additional day staying at the hospital (Fenton 2013: OR = 1.04, 95% CI 1.02–1.06; INTERGROWTH-21st: OR = 1.06, 95% CI 1.03–1.09). From the nutritional factors, for each additional day of delay of full enteral nutrition, the risk of severe PGF increased by 5% or 6% respective to the reference chart (Fenton 2013: OR = 1.05, 95% CI 1.01–1.09; INTERGROWTH-21st: OR = 1.06, 95% CI 1.02–1.10). Late-onset sepsis was recognized as a risk factor for severe PGF (Fenton 2013: OR = 2.14, 95% CI 1.09–4.19; INTERGROWTH-21st: OR = 2.78, 95% CI 1.28–6.05).

No effect modification by infant sex was observed when tested by sample stratification (Supplementary Table S3); however, some effects were attenuated due to sample reduction. Exclusion of SGA infants did not alter the results (Supplementary Table S4). Finally, multinomial logistic regression models were used to estimate relative risk ratios comparing non-severe PGF and severe PGF with reference to neonates without PGF (Supplementary Table S5), but no deviations from the main findings were observed.

**Figure 2:**
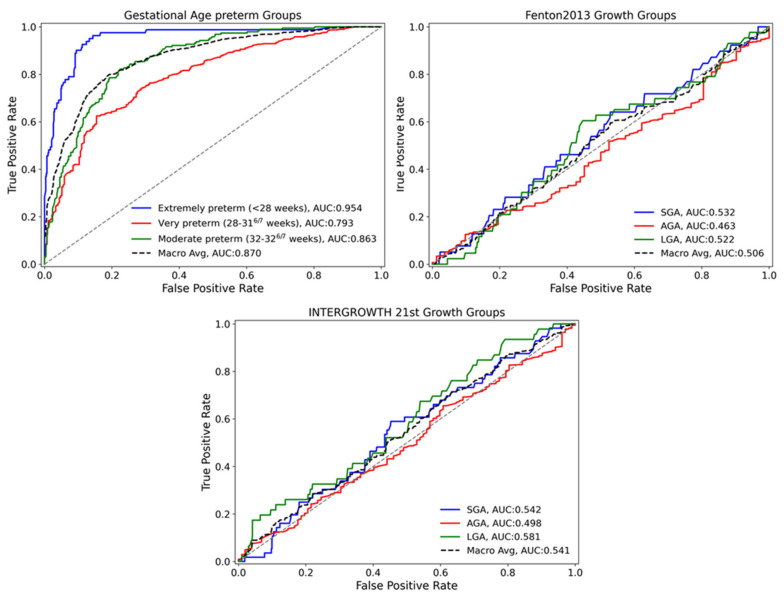
Birth weight for gestational age classification per Fenton 2013/Intergrowth-21st growth curves. Machine learning XGBoost supervised model ROC curves for GA preterm and growth curves groups. Macro-Averaged Area Under the Receiver Operating Characteristic Curve (AUC Macro Avg) evaluates the model’s ability to distinguish between classes across all classification thresholds.

Machine learning models, both supervised and unsupervised, were applied as discussed in the methodology section. To evaluate the performance of various algorithms for classification and prediction, the dataset was analyzed using clinically relevant gestational age groups (extremely preterm (<28 weeks), very preterm (28–31^6/7^ weeks), and moderately preterm (32–32^6/7^ weeks)) as they are clear and relevant to the actual dataset. Among the supervised algorithms tested, XGBoost demonstrated superior classification performance for these gestational age categories and was subsequently utilized to evaluate classifications based on the Fenton 2013 and INTERGROWTH-21st growth curves (SGA, AGA, LGA). Relevant ROC curves for these classifications are depicted in Figure 2 and the corresponding confusion matrices are in Supplementary Figure S1.

Interestingly, while logistic regression analyses consistently highlighted clinical and nutritional factors significantly associated with the risk PGF, findings from the machine learning classification models highlight a different issue. Although these models exhibited high accuracy in classifying infants based purely on gestational age using clinical and nutritional predictors, their predictive accuracy significantly decreased when classifying infants according to both the Fenton 2013 and INTERGROWTH-21st growth curves. Table 4 summarizes the average importance scores (mean ± SD) of the ten most important factors for the gestational age preterm groups shown in Figure 2, evaluated across ten iterations of the best XGBoost supervised models and aims to quantify the relative contribution of each variable to the model’s predictive accuracy. Birth weight (0.279 ± 0.016) was identified as the variable exerting the greatest influence, followed closely by the necessity for respiratory support (0.151 ± 0.021) and administration of aminophylline (0.146 ± 0.006). Supplementary Table S6 has all clinical and nutritional factor average importance scores that influenced all XGBoost models.

## 4. Discussion

In this retrospective study, we analyzed the prevalence of PGF at discharge in neonates born before 33 weeks using two different growth charts. The results of our study demonstrated that, upon discharge, a significantly higher proportion of infants met the criteria for severe and non-severe PGF when assessed using the Fenton 2013 growth curves compared to the INTERGROWTH-21st curves. This finding is consistent with other research [62,63,64], although comparing these studies is challenging due to the significant variations in the definition of EUGR and the emerging term ‘’postnatal growth faltering’’ currently used [65]. In our study, we chose to employ the term “postnatal growth faltering,” which better reflects variations in z-score and allows for a continuous evaluation of weight changes from birth to discharge. Also, in our analysis, severe PGF was defined as a decrease in weight z-score of more than two standard deviations during the neonatal hospital stay, a threshold that has been linked to an increased risk of adverse neurodevelopmental outcomes [9,66]. This approach offers a more accurate representation of growth progression over time, whereas single-time-point assessments reflect only size and may be influenced by factors such as birth weight, limiting their ability to capture true growth trends.

The higher rate of PGF on Fenton 2013 growth charts could be attributed to the fact that these charts are based on optimal intrauterine growth patterns, which set higher growth expectations for preterm infants and, thus, characterize more infants as experiencing PGF. In contrast, the INTERGROWTH-21st charts are based on postnatal growth data, which may reflect the slower growth rates typically seen in preterm infants outside the womb due to complications of prematurity, nutritional challenges, and the overall fragility of these infants [26]. A recent ten-year retrospective study by Starc et al. [67] indicated that the Fenton 2013 growth charts showed a notably higher prevalence of weight loss greater than one SD in very-low-birth-weight neonates compared to the INTERGROWTH-21st charts. However, while the same study found that the prevalence of weight loss greater than two SDs in very-low-birth-weight neonates did not significantly differ across growth charts, our research showed a notably higher prevalence of severe PGF when using the Fenton 2013 charts compared to INTERGROWTH-21st. Although all infants classified with PGF using the INTERGROWTH-21st chart were also identified by the Fenton 2013 curves, the inverse did not apply. This illustrates how different growth standards can lead to varying rates of PGF diagnosis. The choice of reference chart can significantly influence the interpretation of postnatal growth in preterm infants.

The multivariate analysis in our study identified being SGA as a statistically significant factor affecting severe PGF and specifically providing a protective effect. This finding contrasts with our department’s previous retrospective study on preterm neonates born before 32 weeks gestational age, which indicated a negative impact of SGA on cross-sectional EUGR [68]. Nevertheless, this observation aligns with findings from a multicentral, European study by El Rafei et al. [66], which concluded that being SGA at birth had a negative association with EUGR when characterized by percentile measures but a positive association with EUGR when characterized with velocity measures. In our study, PGF is defined dynamically, reflecting changes over time, which justifies the positive association observed. Many neonates who are SGA at birth continue to grow below the 10th percentile and are classified as EUGR at discharge according to cross-sectional standards. However, they do not show a significant decline in z-score that would categorize them as experiencing PGF under the longitudinal definition. Being SGA appears to have a paradoxically protective effect against PGF, likely due to their unique early growth patterns such as reduced initial weight loss and early catch-up growth. Studies have shown that SGA infants typically lose less weight than AGA infants in the first days of life, and some even regain their birth-weight percentile before discharge, which is rarely observed in AGA preterm neonates [69,70]. A study by Molony et al. [70] indicated that preterm SGA neonates are up to four times more likely than their non-SGA peers to improve their weight-for-age z-score during their stay in the NICU. These findings may help explain the protective effect observed in our study.

The Barker hypothesis indicates that an accelerated growth rate may increase the risk of obesity, hypertension, and metabolic syndrome in later life. However, recent evidence does not support these associations in preterm SGA infants. In fact, these infants frequently demonstrate comparable—or sometimes even better—long-term health outcomes than those born being AGA. A recent meta-analysis of 253,810 singletons specifically found that gestational age, rather than birth weight, is more important for body size in infancy, with preterm individuals showing similar mean BMI to term-born peers by adolescence [71]. Although prematurity is associated with an increased risk of overweight in adolescence and cardiovascular risk later in life, overall obesity and cardiovascular disease risk remains strongly influenced by lifetime environmental factors [71,72]. In fact, recent critiques have questioned the credibility of the developmental origins of health and disease hypothesis, citing biases and excessive adjustments to body weight statistics at the time of outcome assessment in the original studies [73].

In our research, the length of hospitalization strongly correlated with severe PGF across both growth charts, with each additional hospital day increasing severe PGF risk. This could be attributed to the numerous complications of prematurity, which could extend their hospital stay, along with various clinical factors. Preterm neonates with complications of prematurity experience high energy expenditure, chronic stress, and fluid restrictions, all of which contribute to a negative nitrogen balance, resulting in a higher incidence of PGF [74,75]. However, our multivariate analysis could not support these correlations except that the late-onset sepsis was recognized as a risk factor for severe PGF in both growth charts. Flannery et al. [76], in a study of nearly 700 very-low-birth-weight infants born at <32 weeks’ gestation, reported that those with sepsis exhibited a significantly greater decline in weight z-scores compared to infants without sepsis, with growth faltering becoming evident by three weeks post-infection and persisting until NICU discharge. Sepsis may contribute to PGF by increasing energy expenditure and inducing a catabolic state that impairs lean mass and fat accumulation, while also disrupting IGF-1 levels essential for early postnatal growth [76,77].

Our research highlighted that while both early initiation and timely achievement of full enteral feeding were associated with reduced PGF, it was the attainment of full enteral feeding that played a key role in reducing the risk of severe PGF—a finding consistent with previous studies [78,79]. A prominent American multicenter study by Stevens et al. [79], involving nearly 5500 neonates born under 31 weeks of gestational age, demonstrated that improved nutritional practices, particularly earlier initiation and achievement of full enteral feeding, significantly reduced the drop in the z-scores between birth and discharge weights. However, the benefits of enteral nourishment for preterm infants are tempered by gastrointestinal challenges ranging from feeding intolerance to surgical necrotizing enterocolitis, complicating the initiation and advancement of feeding [80]. Despite these challenges, early and progressive enteral feeding is essential for delivering key nutrients, promoting intestinal maturation, supporting a healthy microbiome, reducing inflammation, and enhancing neurodevelopment [81]. It is important to note that in our study, the prevalence of severe and non-severe PGF has been decreasing over the years. This finding is in accordance with the results of a multicenter study in California involving nearly 26,000 very-low-birth-weight infants born at 22 to 32 weeks GA, which reported a significant decrease in weight z-scores by 0.112 between birth and discharge during the study period [82]. Similarly, in a study by González-García [22] comparing two periods, 2002–2006 and 2013–2017, very-low-birth-weight preterm neonates showed a lower prevalence of PGF and an increased growth rate in the first 28 days in the second period compared to the first. This improvement can be attributed to the adoption of more liberal nutritional practices based on newer guidelines, including the earlier initiation of enteral feeding, advancing enteral feeding more rapidly with higher volumes (30 mL/kg/day compared to 15–20 mL/kg/day), and achieving full enteral feeding sooner. Our study also explored the role of PN duration in the development of PGF; even though longer PN duration was correlated positively with PGF, it was not identified as a risk factor in the multivariate analysis for either growth chart, suggesting that the quality of nutrient intake—particularly protein and energy content during the transitional phase when PN is reduced and enteral feeds increased—may have a greater impact on growth outcomes [83,84].

The present study applied supervised machine learning methods, notably XGBoost, to classify preterm neonates into distinct gestational age groups, effectively identifying clinical and nutritional risk factors associated with PGF, consistent with recent literature validating machine learning’s superior predictive capabilities in neonatal growth outcomes [85,86]. Interestingly, when classifications based on internationally recognized growth curves (Fenton 2013 and INTERGROWTH-21st) were evaluated in our Greek cohort, a notable decline in predictive accuracy was observed, aligning with previous research highlighting discrepancies in PGF rates depending on the growth standard applied [64,68]. This discrepancy likely arises because these international growth curves were not specifically developed for or validated in the Greek neonatal population. Consequently, their limited predictive power underscores the inherent mismatch when applying international standards that fail to capture the distinct growth patterns of neonates in Greece, suggesting a pressing need for nationally specific growth references to ensure accurate identification and management of PGF in this population. Out team recently demonstrated that the prevalence of PGF among Greek preterm neonates significantly differed between these international growth references, underlining their limited applicability to local populations [68]. Similarly, studies from other Mediterranean and global cohorts underscore that international standards do not adequately reflect specific population growth trajectories, leading to potential misclassification and clinical mismanagement [87,88]. Consequently, our findings support the critical need for nationally derived, population-specific growth references in Greece. Integrating such tailored references with advanced machine learning methods could substantially enhance early risk identification and targeted nutritional interventions, ultimately improving neonatal growth outcomes and reducing long-term morbidities in vulnerable preterm populations [89].

There are several limitations to our study. First, as only weight at birth and at discharge were registered in the database, information on growth patterns such as weight loss during the first few days after birth or weight at specified timing (i.e., PMA 36 weeks) was not provided. Additionally, due to the retrospective nature of our study, it was challenging to collect body composition measurements, and as such data were not consistently recorded in the past. Concerning dietary information, our study did not encompass the progression of enteral feeding volume, the specific type of enteral feeding (such as breast milk or formula feeding), and the composition of parenteral nutrition in terms of protein, lipids, and energy. Additionally, we did not include information on maternal medical history during pregnancy, such as diabetes mellitus and preeclampsia, preventing us from incorporating these data into our study. The other limitation of this study was the lack of long-term outcomes like growth, metabolism, and neurodevelopment, which are closely linked to PGF [90]. According to Pampanini et al. [91], a significant proportion of prematurely born children experiencing severe PGF exhibit growth retardation in childhood, highlighting the necessity for close clinical monitoring of their growth during childhood and adulthood. Neurodevelopment is a crucial factor in defining PGF and guiding necessary interventions. Zozaya et al. [10] described that a decrease in the weight z-score by one point using the Fenton 2013 charts correlates with a significant reduction in the Mental Development Index (MDI) at 24 months of corrected gestational age in preterm infants under 34 weeks. However, other studies have reported no link between PGF and poor neurodevelopmental outcomes [23,92]. Further research using varied definitions of PGF and multiple neurodevelopmental assessments is warranted. It is important to note that the accuracy of the INTERGROWTH-21st growth curves in evaluating infants born before 33 weeks’ gestation is questionable due to the limited contribution of preterm infants in developing these curves. Therefore, additional research using extensive multicenter population-based data is essential to explore the efficacy of employing these postnatal growth curves for preterm infants with the smallest gestational ages [93].

## 5. Conclusions

In conclusion, our study confirms that severe and non-severe PGF significantly affects premature neonates, although this trend has declined over the years covered by the study. This encouraging trend may be linked to the implementation of a quicker establishment of full enteral feeds, which significantly reduces the risk of severe PGF. Notably, Fenton 2013 curves indicated a higher percentage of both severe and non-severe PGF compared to INTERGROWTH-21st, highlighting the need for standardizing growth assessment tools. Factors that seem to be associated with severe PGF in our study included prolonged hospitalization and late onset sepsis, whereas being SGA appeared to have a protective effect against severe PGF. Further research is essential to determine the most suitable growth curve for evaluating preterm infant growth. It is crucial to prioritize high-quality studies using standardized neurodevelopmental tools beyond 2 years of age which may be more predictive of later cognitive outcomes [94,95]. Finally, developing population-specific growth references in Greece, enhanced by machine learning, could improve growth monitoring and nutritional management in preterm infants.

## Figures and Tables

**Table 1:** Study population characteristics (*n* = 650).

Characteristic	*N* (%)	Mean (SD)
**Infant sex**		
Male	350 (53.8%)	
Female	300 (46.2%)	
**Gestational age at birth** (weeks)	650 (100%)	30.4 (1.9)
**Prematurity**		
<28 weeks	80 (12.3%)	
28–31^6/7^ weeks	379 (58.3%)	
32–32^6/7^ weeks	191 (29.4%)	
**Birth weight** (grams)	650 (100%)	1475.9 (399.4)
**Multiple pregnancy**		
No	361 (55.6%)	
Yes	288 (44.4%)	
**Type of delivery**		
Caesarean	574 (88.3%)	
Vaginal	76 (11.7%)	
**Postmenstrual age at discharge** (weeks)	650 (100%)	37.1 (2.7)
**Weight at discharge** (grams)	650 (100%)	2405.5 (386.2)
**PGF by Fenton 2013**		
Normal (ΔZ weight ≥ −1)	273 (42.4%)	
Non-severe PGF (−2 ≤ ΔZ weight < −1)	277 (43.0%)	
Severe PGF (ΔZ weight < −2)	94 (14.6%)	
**PGF by INTERGROWTH-21st**		
Normal (ΔZ weight ≥ −1)	420 (65.2%)	
Non-severe PGF (−2 ≤ ΔZ weight < −1)	158 (24.5%)	
Severe PGF (ΔZ weight < −2)	66 (10.3%)	

PGF, postnatal growth faltering; ΔZ weight, difference in standard deviation scores of weight from birth to discharge. **Note:** Bolded text indicates major category headings (e.g., Infant sex, Type of delivery) to distinguish them from their respective subcategories.

**Table 2:** Main clinical and nutritional factors associated with PGF for Fenton 2013 and INTERGROWTH-21st growth charts.

	Fenton 2013 *				INTERGROWTH-21st *			
	Normal	Non-Severe PGF	Severe PGF	*p*-Value	Normal	Non-Severe PGF	Severe PGF	*p*-Value
	*N* (%)	*N* (%)	*N* (%)		*N* (%)	*N* (%)	*N* (%)	
Male	135 (39.1%)	159 (46.1%)	51 (14.8%)	0.173	202 (58.6%)	104 (30.1%)	39 (11.3%)	<0.001
Female	138 (46.2%)	118 (39.5%)	43 (14.4%)		218 (72.9%)	54 (18.1%)	27 (9.0%)	
Gestational age at birth	31.3 (2.0)	31.0 (2.3)	28.0 (3.0)	<0.001	31.1 (2.0)	30.3 (2.6)	28.0 (3.0)	<0.001
<28 weeks	21 (26.9%)	19 (24.4%)	38 (48.7%)	<0.001	28 (35.9%)	20 (25.6%)	30 (38.5%)	<0.001
28–31^6/7^ weeks	157 (41.6%)	171 (45.4%)	49 (13.0%)		245 (65.0%)	99 (26.3%)	33 (8.8%)	
32–32^6/7^ weeks	95 (50.3%)	87 (46.0%)	7 (3.7%)		147 (77.8%)	39 (20.6%)	3 (1.6%)	
Birth weight (grams)	1530 (500)	1560 (540)	1160 (580)	<0.001	1565 (547.5)	1480 (530)	1150 (510)	<0.001
SGA								
No	248 (41.0%)	266 (44.0%)	91 (15.0%)	0.018	374 (63.6%)	152 (25.9%)	62 (10.5%)	0.018
Yes	25 (64.1%)	11 (28.2%)	3 (7.7%)		46 (82.2%)	6 (10.7%)	4 (7.1%)	
Type of delivery								
Caesarean	249 (43.8%)	243 (42.8%)	76 (13.4%)	0.026	373 (65.7%)	143 (25.2%)	52 (9.2%)	0.037
Vaginal	24 (31.6%)	34 (44.7%)	18 (23.7%)		47 (61.8%)	15 (19.7%)	14 (18.4%)	
Multiple pregnancy								
No	136 (38.2%)	157 (44.1%)	63 (17.7%)	0.014	223 (62.6%)	87 (24.4%)	46 (12.9%)	0.043
Yes	136 (47.4%)	120 (41.8%)	31 (10.8%)		196 (68.3%)	71 (24.7%)	20 (7.0%)	
	**Median (IQR)**	**Median (IQR)**	**Median (IQR)**		**Median (IQR)**	**Median (IQR)**	**Median (IQR)**	
Hospitalization (days)	36 (27–45)	41.0 (30–55)	76.5 (63–97)	<0.001	36.5 (27–46)	46.5 (36–61)	83.5 (69–104)	<0.001
Parenteral nutrition (days)	6 (1–11)	7 (1–13)	19 (9–35)	<0.001	6 (0–11)	9 (5–16)	22,5 (11–36)	<0.001
Initiation of enteral nutrition (day of life)	2 (2–4)	4 (2–5)	6 (3–10)	<0.001	2 (2–4)	4 (3–7)	7 (3–11)	<0.001
Full enteral nutrition (day of life)	9 (6–12)	12 (8–19)	25 (16–34)	<0.001	9 (6–13)	16 (11–23)	27 (18–38)	<0.001
Oxygen therapy (days)	2.0 (0–4)	3.0 (1–6)	15.5 (3–38)	<0.001	2.0 (0–5)	4.5 (2–8)	22.5 (6–44)	<0.001
Respiratory support (days)	3.0 (0–7)	3.0 (0–8)	15.0 (3–37)	<0.001	2.0 (0–7)	5.0 (1–13)	18.0 (5–38)	<0.001
Mechanical ventilation (days)	0.0 (0–1)	1.0 (0–2)	4.0 (1–14)	<0.001	0.0 (0–1)	1.0 (0–4)	5.0 (1–14)	<0.001
Non-invasive ventilation (days)	2.0 (0–6)	1.0 (0–6)	7.0 (1–24)	<0.001	1.0 (0–6)	2.0 (0–9)	9.5 (1–23)	<0.001

Abbreviations: PGF, postnatal growth faltering; SGA, small for gestational age. * Descriptive measures of continuous factors are presented as median and interquartile range (IQR), whereas categorical factors are presented as frequencies and corresponding percentages. *p*-values obtained from Kruskal–Wallis non-parametric test for distribution of continuous variables and Pearson’s chi-square test for categorical variables.

**Table 3:** Effect of clinical and nutritional factors on the risk of severe PGF using Fenton 2013 and INTERGROWTH-21st growth charts.

Factor	Fenton 2013 OR (95% CI)	INTERGROWTH-21st OR (95% CI)
Gestational age (weeks)	0.81 (0.64, 1.03)	1.01 (0.77, 1.32)
Hospitalization (days)	1.04 (1.02, 1.06)	1.06 (1.03, 1.09)
Respiratory support(days)	0.97 (0.93, 1.00)	0.98 (0.95, 1.02)
Oxygen therapy(days)	1.01 (0.98, 1.04)	0.99 (0.96, 1.02)
Parenteral nutrition(days)	1.01 (0.98, 1.05)	1.01 (0.97, 1.05)
Enteral nutrition (day of life)	1.06 (0.98, 1.14)	1.04 (0.95, 1.13)
Full enteral nutrition (day of life)	1.05 (1.01, 1.09)	1.06 (1.02, 1.10)
Female sex	1.06 (0.57, 1.99)	0.66 (0.30, 1.46)
Vaginal delivery	0.88 (0.35, 2.20)	0.58 (0.20, 1.71)
Multiple pregnancy	0.83 (0.43, 1.61)	0.58 (0.25, 1.36)
SGA	0.11 (0.01, 0.92)	0.10 (0.02, 0.60)
Bronchopulmonary dysplasia	0.90 (0.30, 2.66)	0.39 (0.11, 1.38)
Late-onset sepsis	2.14 (1.09, 4.19)	2.78 (1.28, 6.05)
Anemia	1.04 (0.45, 2.40)	1.88 (0.64, 5.51)

Abbreviations: PGF, postnatal growth faltering; SGA, small for gestational age; odds ratios (ORs) and corresponding 95% confidence intervals (95% CIs) were obtained from binary logistic regression models comparing children with severe PGF to all other children. Models included adjustment for variables that were significantly associated with the outcome in bivariate analyses (*p* < 0.05) and had expected frequencies above 5% across all three levels of the outcome.

**Table 4:** Top clinical and nutritional factors that influenced XGBOOST supervised models for the gestational-age preterm groups. Average importance scores (mean ± SD) of ten most important factors, evaluated across ten CV iterations of the XGBoost supervised model. Best model shown in Figure 2 used the top four.

Factor	Average Significance Mean ± SD
Birth weight (grams)	0.279 ± 0.016
Respiratory support (days)	0.151 ± 0.021
Aminophylline administration (days)	0.146 ± 0.006
Caffeine administration (days)	0.100 ± 0.014
Red blood cell transfusion (count)	0.069 ± 0.011
Oxygen therapy (days)	0.067 ± 0.010
Enteral nutrition initiation (days)	0.062 ± 0.015
Non-invasive ventilation (days)	0.059 ± 0.019
Multiple birth	0.042 ± 0.007
Parenteral nutrition initiation (days)	0.025 ± 0.012

## Data Availability

The datasets presented in this article are not readily available because they contain patient data that are not for public use due to European data protection laws (GDPR). Requests to access the datasets should be directed to corresponding author.

## Supplementary Materials

The following supporting information can be downloaded at: https://www.mdpi.com/article/10.3390/nu17101726/s1, Table S1. Quality Metrics for XGBoost Models. Table S2. Main clinical and nutritional factors associated with severe PGF by Fenton 2013 and INTERGROWTH-21st growth charts. Table S3. Effect of clinical and nutritional factors on the risk of severe PGF using Fenton 2013 and INTERGROWTH-21st reference charts, stratified by infant sex. Table S4. Effect of clinical and nutritional factors on the risk of severe PGF using Fenton 2013 and INTERGROWTH-21st reference charts, excluding SGA infants. Table S5. Clinical and nutritional factors associated with severity of PGF by Fenton 2013 and INTERGROWTH-21st growth charts. Table S6. Clinical and nutritional factors that influenced XGBOOST supervised models. Average importance scores (mean ± SD) of ten most important factors, evaluated across ten CV iterations of the XGBoost supervised model. Figure S1. Confusion matrices for each of the XGBoost results. For Gestational Age Preterm groups 1 is Extremely preterm (<28 weeks), 2 is Very preterm (28-31^6/7^ weeks) and 3 is Moderate preterm (32–32^6/7^ weeks).
