# Supplementary material for: Evaluating Prevalence of Preterm Postnatal Growth Faltering Using Fenton 2013 and INTERGROWTH-21st Growth Charts with Logistic and Machine Learning Models
Source: Nutrients. 2025 May 20;17(10):1726. doi: 10.3390/nu17101726 (PMC12114127; doi:10.3390/nu17101726)
Supplement: Supplementary file 1 [file nutrients-17-01726-s001.zip › nutrients-3598546-supplementary.pdf]

nutrients

MDPI

Evaluating Preterm Postnatal Growth Faltering Prevalence Using Fenton2013 and INTERGROWTH-21st Growth Charts with Logistic and Machine Learning Models

Supplementary file Contents

Supplementary Table 1 Quality Metrics for XGBoost Models 2
Supplementary Table 2 Main clinical and nutritional factors associated with severe PGF by Fenton2013 and INTERGROWTH-21st growth charts.. 3
Supplementary Table 3 Effect of clinical and nutritional factors on the risk of severe PGF using Fenton2013 and INTERGROWTH-21st reference charts, stratified by infant sex. 4
Supplementary Table 4 Effect of clinical and nutritional factors on the risk of severe PGF using Fenton2013 and INTERGROWTH-21st reference charts, excluding SGA infants. 5
Supplementary Table 5 Clinical and nutritional factors associated with severity of PGF by Fenton2013 and INTERGROWTH-21st growth charts. 6
Supplementary Table 6 Clinical and nutritional factors that influenced XGBOOST supervised models. Average importance scores (mean ± SD) of ten most important factors, evaluated across ten CV iterations of the XGBoost supervised model……………………………………………………………7
Supplementary Figure 1. Confusion matrices for each of the XGBoost results. For Gestational Age Preterm groups 1 is Extremely preterm (<28 weeks) , 2 is Very preterm (28-31 6/7 weeks) and 3 is Moderate preterm (32-32 6/7 weeks)……………………………………………....................................8

Supplementary Table S1. Quality Metrics for XGBoost Models.

Metrics	Preterm	Fenton2013	INTERGROWTH-21st
**Accuracy**	0.709	0.648	0.608
**WA_Precision Score**	0.737	0.761	0.732
**WA_Recall Score**	0.709	0.648	0.608
**WA_F1 Score**	0.711	0.698	0.658
**Cohen_kappa Score**	0.521	-0.03	0.034
**MCC Score**	0.532	-0.033	0.037
**Hamming Loss**	0.291	0.352	0.392
**AUC Macro Avg**	0.87	0.506	0.541

Supplementary Table S2. Main clinical and nutritional factors associated with severe PGF by Fenton2013 and INTERGROWTH-21st growth charts.

	Fenton2013*			INTERGROWTH-21st		
	Other	Severe PGF	p-value	Other	Severe PGF	p-value
	N (%)	N (%)		N (%)	N (%)	
**Male**	294 (85.2)	51 (14.8)	0.886	306 (88.7)	39 (11.3)	0.343
**Female**	256 (85.6)	43 (14.4)		272 (91.0)	27 (9.0)	
**Gestational Age at birth**	31.0 (2.1)	28.0 (3.0)	<0.001	31 (2.1)	28.0 (3.0)	<0.001
**<28 weeks**	40 (51.3)	38 (48.7)	<0.001	48 (61.5)	30 (38.5)	<0.001
**28-31 6/7 weeks**	328 (87.0)	377 (13.0)		344 (91.3)	377 (8.8)	
**32-32 6/7 weeks**	182 (96.3)	189 (3.7)		186 (98.4)	189 (1.6)	

 https://doi.org/10.3390/xxxxx

**SGA**						
**No**	514 (85.0)	91 (15.0)	0.208	541 (89.4)	64 (10.5)	0.277
**Yes**	36 (92.3)	3 (7.7)		37 (94.9)	2 (7.1)	
**Type of delivery**						
**Caesarean**	492 (86.6)	76 (13.4)	0.017	516 (90.9)	52 (9.2)	0.012
**Vaginal**	58 (76.3)	18 (23.7)		62 (81.6)	14 (18.4)	
**Multiple pregnancy**						
**No**	293 (82.3)	63 (17.7)	0.014	310 (87.1)	46 (12.9)	0.013
**Yes**	256 (89.2)	31 (10.8)		267 (93.0)	20 (7.0)	
	**Median (IQR)**	**Median (IQR)**		**Median (IQR)**	**Median (IQR)**	
**Hospitalization (days)**	38 (28-50)	76.5 (63-97)	<0.001	39 (29-52)	83.5 (69-104)	<0.001
**Parenteral Nutrition(*days*)**	6 (1-11)	19 (9-35)	<0.001	6 (1-12)	22,5 (11-36)	<0.001
**Enteral Nutrition (*day of life*)**	3 (2-5)	6 (3-10)	<0.001	3 (2-5)	7 (3-11)	<0.001
**Full Enteral Nutrition (*day of life*)**	10 (7-16)	25 (16-34)	<0.001	10 (7-17)	27 (18-38)	<0.001
**Oxygen Therapy (days)**	3 (1-6)	15.5 (3-38)	<0.001	2 (1-6)	22.5 (6-44)	<0.001
**Respiratory Support (days)**	3 (0-8)	15 (3-37)	<0.001	3 (0-8)	18 (5-38)	<0.001
**Mechanical Ventilation (days)**	1 (0-2)	4 (1-14)	<0.001	0 (0-2)	5 (1-14)	<0.001
**Non-Invasive Ventilation (days)**	1 (0-6)	7 (1-24)	<0.001	1.5 (0-6)	9.5 (1-23)	<0.001

Supplementary Table S3. Effect of clinical and nutritional factors on the risk of severe PGF using Fenton2013 and INTERGROWTH-21st reference charts, stratified by infant sex.

	**Fenton2013**			**INTERGROWTH-21st**		
	**OR**	**95% CI**		**OR**	**95% CI**	
**BOYS**						
Gestational age (weeks)	0.57	0.37	0.89	0.81	0.51	1.27
Hospitalization (days)	1.05	1.00	1.09	1.06	1.01	1.10
Respiratory Support(days)	0.91	0.86	0.97	0.95	0.90	1.00
Oxygen Therapy(days)	1.04	0.99	1.09	1.04	0.99	1.10
Parenteral Nutrition(days)	1.01	0.95	1.08	0.98	0.91	1.05
Enteral Nutrition (day of life)	1.04	0.90	1.19	0.99	0.87	1.13
Full Enteral Nutrition (day of life)	1.05	0.99	1.13	1.08	1.01	1.16
Vaginal delivery	0.48	0.11	2.05	0.29	0.06	1.36
Multiple pregnancy	0.73	0.26	2.05	0.50	0.15	1.69
SGA	1.00			1.00		
Bronchopulmonary dysplasia	2.65	0.58	12.13	0.61	0.12	3.17
Late-onset sepsis	2.45	0.82	7.27	2.27	0.70	7.32
Anaemia	0.89	0.23	3.42	2.94	0.50	17.16
**GIRLS**						
Gestational age (weeks)	1.04	0.76	1.44	1.49	0.98	2.26
Hospitalization (days)	1.05	1.02	1.08	1.11	1.06	1.16
Respiratory Support(days)	1.00	0.96	1.05	1.05	0.98	1.11
Oxygen Therapy(days)	0.99	0.95	1.04	0.93	0.88	0.98
Parenteral Nutrition(days)	1.00	0.96	1.05	1.00	0.93	1.08
Enteral Nutrition (day of life)	1.05	0.95	1.17	1.09	0.95	1.24
Full Enteral Nutrition (day of life)	1.05	1.00	1.10	1.05	0.99	1.12
Vaginal delivery	1.24	0.34	4.47	0.94	0.16	5.54
Multiple pregnancy	0.99	0.38	2.57	1.21	0.28	5.17
SGA	0.17	0.02	1.71	0.07	0.01	0.76
Bronchopulmonary dysplasia	0.31	0.05	1.87	0.13	0.01	1.35

Late-onset sepsis	1.81	0.67	4.83	3.52	0.90	13.82
Anaemia	1.07	0.34	3.41	1.72	0.33	8.91

Abbreviations: PGF, Postnatal growth faltering; SGA, small for gestational age; OR: Odds Ratio; CI: Confidence Interval. Odds ratios and corresponding confidence intervals obtained from binary logistic regression models entering simultaneously the factors found to be correlated ($p<0.05$) at the bivariate comparisons and to have expected frequencies above 5% in all levels of the outcome.

Supplementary Table S4. Effect of clinical and nutritional factors on the risk of severe PGF using Fenton2013 and INTERGROWTH-21st reference charts, excluding SGA infants.

	**Fenton2013**			**INTERGROWTH-21st**		
	**OR**	**95% CI**		**OR**	**95% CI**	
**BOYS**						
Gestational age (weeks)	0.77	0.60	0.98	0.97	0.73	1.28
Hospitalization (days)	1.03	1.01	1.05	1.05	1.03	1.08
Respiratory Support(days)	0.96	0.93	1.00	0.98	0.95	1.02
Oxygen Therapy(days)	1.01	0.98	1.04	1.00	0.97	1.03
Parenteral Nutrition(days)	1.02	0.98	1.05	1.01	0.97	1.05
Enteral Nutrition (day of life)	1.06	0.98	1.15	1.04	0.96	1.13
Full Enteral Nutrition (day of life)	1.05	1.01	1.09	1.07	1.02	1.11
Infant sex	1.07	0.57	2.02	0.63	0.28	1.42
Vaginal delivery	0.92	0.37	2.29	0.60	0.21	1.76
Multiple pregnancy	0.85	0.43	1.65	0.59	0.25	1.39
Bronchopulmonary dysplasia	0.89	0.30	2.67	0.39	0.11	1.40
Late-onset sepsis	1.97	0.99	3.88	2.32	1.05	5.13
Anaemia	1.19	0.52	2.75	2.40	0.79	7.35

Abbreviations: PGF, Postnatal growth faltering; SGA, small for gestational age; OR: Odds Ratio; CI: Confidence Interval. Odds ratios and corresponding confidence intervals obtained from binary logistic regression models entering simultaneously the factors found to be correlated ($p<0.05$) at the bivariate comparisons and to have expected frequencies above 5% in all levels of the outcome.

Scupplementary Table S5. Clinical and nutritional factors associated with severity of PGF by Fenton2013 and INTERGROWTH-21st growth charts. .

	**Normal**	**Non severe PGF**			**Severe PGF**		
**Predictors**		**Relative Risk**	**95% CI**		**Relative Risk**	**95% CI**	
		**FENTON2013**					
Gestational age (weeks)	Ref.	1.05	0.88	1.25	0.85	0.63	1.16
Hospitalization (days)	Ref.	1.02	1.00	1.04	1.06	1.02	1.10
Respiratory Support(days)	Ref.	0.98	0.94	1.01	0.95	0.91	0.99
Oxygen Therapy(days)	Ref.	0.99	0.95	1.03	1.00	0.96	1.04
Parenteral Nutrition(days)	Ref.	0.94	0.91	0.98	0.98	0.94	1.02
Enteral Nutrition (day of life)	Ref.	1.17	1.05	1.31	1.19	1.04	1.37
Full Enteral Nutrition (day of life)	Ref.	1.10	1.06	1.15	1.12	1.07	1.18
Female sex	Ref.	0.63	0.44	0.90	0.80	0.39	1.60
Vaginal delivery	Ref.	1.38	0.72	2.65	1.12	0.34	3.73
Multiple pregnancy	Ref.	0.73	0.50	1.08	0.66	0.33	1.32
SGA	Ref.	0.31	0.13	0.74	0.05	0.01	0.41
Bronchopulmonary dysplasia	Ref.	2.95	0.99	8.76	1.90	0.46	7.88
Late-onset sepsis	Ref.	1.40	0.77	2.56	2.58	1.09	6.10
Anaemia	Ref.	0.72	0.40	1.30	0.83	0.33	2.09
		**INTERGROWTH-21st**					
Gestational age (weeks)	Ref.	0.95	0.79	1.15	0.97	0.70	1.36

Hospitalization (days)	Ref.	1.03	1.01	1.05	1.08	1.03	1.13
Respiratory Support(days)	Ref.	0.98	0.95	1.01	0.97	0.93	1.01
Oxygen Therapy(days)	Ref.	1.00	0.97	1.03	0.99	0.95	1.04
Parenteral Nutrition(days)	Ref.	0.97	0.93	1.01	0.99	0.95	1.03
Enteral Nutrition (day of life)	Ref.	1.09	0.99	1.19	1.09	0.96	1.24
Full Enteral Nutrition (day of life)	Ref.	1.09	1.05	1.14	1.12	1.06	1.18
Female sex	Ref.	0.38	0.24	0.59	0.38	0.16	0.91
Vaginal delivery	Ref.	0.53	0.26	1.09	0.41	0.11	1.47
Multiple pregnancy	Ref.	0.93	0.61	1.44	0.58	0.24	1.37
SGA	Ref.	0.14	0.05	0.46	0.04	0.01	0.26
Bronchopulmonary dysplasia	Ref.	1.06	0.36	3.08	0.39	0.08	1.77
Late-onset sepsis	Ref.	1.64	0.93	2.91	3.67	1.52	8.87
Anaemia	Ref.	0.74	0.41	1.34	1.49	0.48	4.65

Abbreviations: PGF, Postnatal growth faltering; SGA, small for gestational age; OR: Odds Ratio; CI: Confidence Interval. Odds ratios and corresponding confidence intervals obtained from multinomial logistic regression models including factors found to be correlated ($p<0.05$) at the bivariate comparisons previously described and to have expected frequencies above 5% in all three levels of the outcome..

Supplementary Table S6. Clinical and nutritional factors that influenced XGBOOST supervised models. Average importance scores (mean ± SD) of ten most important factors, evaluated across ten CV iterations of the XGBoost supervised model.

**Factor**	**Preterm (mean ± SD)**	**FENTON2013 (mean ± SD)**	**INTERGROWTH-21st (mean ± SD)**
Birthweight	0.254 ± 0.026	0.205 ± 0.018	0.218 ± 0.009
Respiratory Support(days)	0.128 ± 0.020	0.103 ± 0.016	0.098 ± 0.007
Days of aminophylline administration	0.126 ± 0.017	0.086 ± 0.010	0.078 ± 0.009
Days of caffeine administration	0.084 ± 0.011	0.083 ± 0.012	0.067 ± 0.009
Transfusion RBC (doses)	0.065 ± 0.011	0.061 ± 0.012	0.063 ± 0.011
Enteral Nutrition (day of life)	0.052 ± 0.015	0.051 ± 0.012	0.061 ± 0.010
Oxygen Therapy(days)	0.052 ± 0.012	0.051 ± 0.011	0.043 ± 0.006
Duration of non - invasive ventilation (days)	0.050 ± 0.016	0.047 ± 0.011	0.039 ± 0.005
Number of neonates born	0.038 ± 0.007	0.041 ± 0.011	0.039 ± 0.013
Initiation of parenteral nutrition (day of life)	0.028 ± 0.018	0.039 ± 0.009	0.038 ± 0.004
IVH	0.020 ± 0.012	0.029 ± 0.007	0.031 ± 0.006
Apnoea	0.019 ± 0.003	0.027 ± 0.005	0.028 ± 0.006
Sex Female	0.017 ± 0.007	0.026 ± 0.007	0.025 ± 0.007
Early Onset Infection	0.016 ± 0.006	0.024 ± 0.007	0.019 ± 0.005
Duration of MV (days)	0.008 ± 0.005	0.016 ± 0.004	0.019 ± 0.004

Surfactant (doses)	0.006 ± 0.004	0.016 ± 0.005	0.017 ± 0.004
ROP	0.005 ± 0.004	0.015 ± 0.006	0.014 ± 0.005
Late-onset Septicemia(>72 hrs)	0.005 ± 0.005	0.014 ± 0.004	0.014 ± 0.007
Late-onset Infection (>72hrs)	0.005 ± 0.007	0.013 ± 0.007	0.014 ± 0.003
Hypotension	0.005 ± 0.004	0.012 ± 0.004	0.013 ± 0.004
Dopamine	0.005 ± 0.006	0.011 ± 0.004	0.013 ± 0.005
Dobutamine	0.004 ± 0.004	0.009 ± 0.006	0.013 ± 0.005
PVL	0.003 ± 0.004	0.004 ± 0.003	0.011 ± 0.004
Vaginal Delivery	0.003 ± 0.004	0.004 ± 0.003	0.006 ± 0.003
Theophylline	0.002 ± 0.004	0.004 ± 0.004	0.006 ± 0.005
PDA	0.001 ± 0.002	0.003 ± 0.002	0.004 ± 0.003
Pneumothorax	0.000 ± 0.000	0.002 ± 0.003	0.002 ± 0.003
BPD	0.000 ± 0.000	0.000 ± 0.000	0.000 ± 0.001
NEC	0.000 ± 0.000	0.000 ± 0.000	0.000 ± 0.000
Anemia	0.000 ± 0.000	0.000 ± 0.000	0.000 ± 0.000
EOSept_	0.000 ± 0.000	0.000 ± 0.000	0.000 ± 0.000

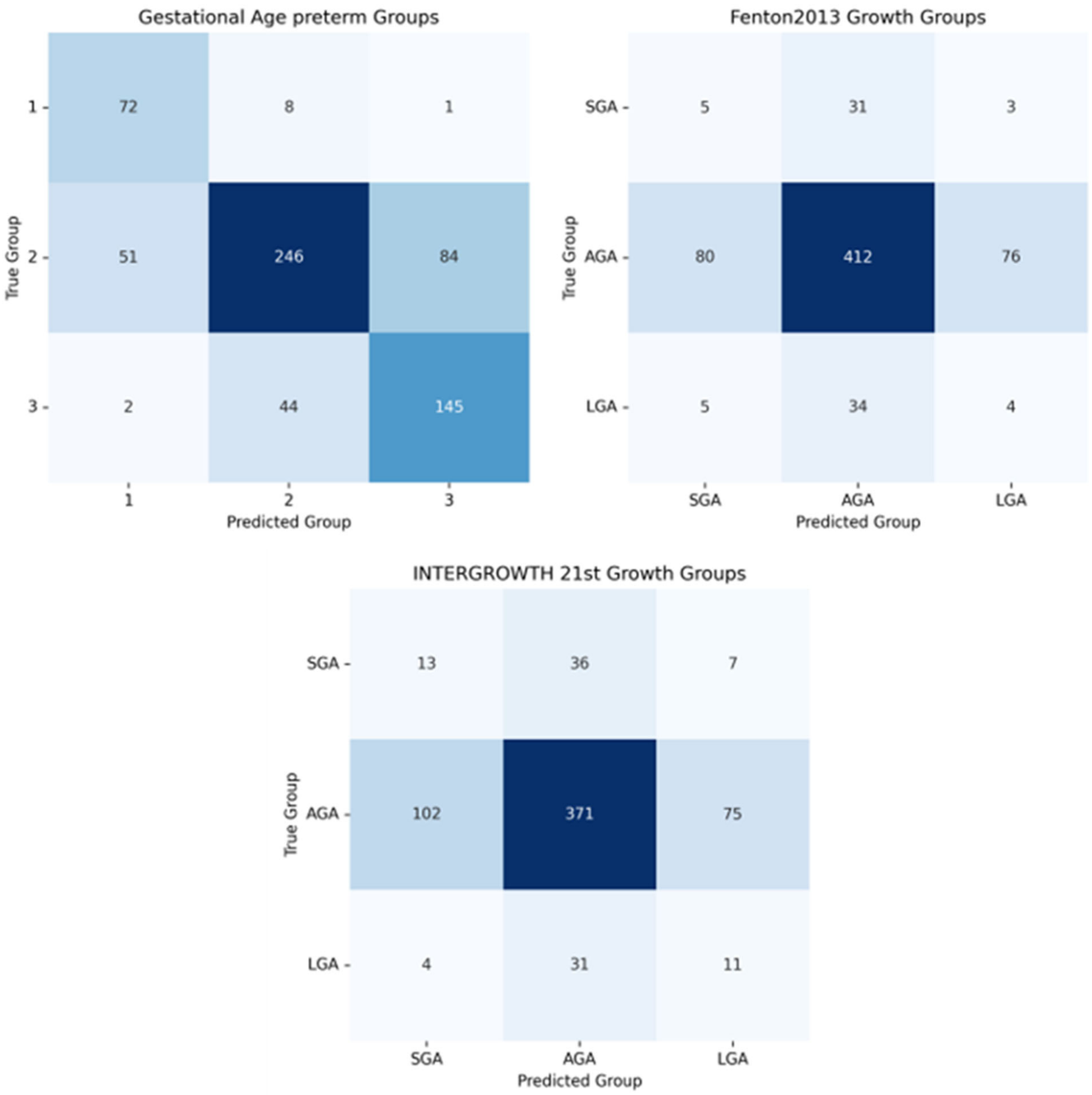

Supplementary Figure S1. Confusion matrices for each of the XGBoost results. For Gestational Age Preterm groups 1 is Extremely preterm (<28 weeks), 2 is Very preterm (28-31 6/7 weeks) and 3 is Moderate preterm (32-32 6/7 weeks).
